# Supplementary material for: Platelet-Derived Extracellular Vesicles as Lipid Carriers in Severe Allergic Inflammation
Source: Int J Mol Sci. 2023 Aug 12;24(16):12714. doi: 10.3390/ijms241612714 (PMC10454366; doi:10.3390/ijms241612714)
Supplement: Supplementary file 1 [file ijms-24-12714-s001.zip › ijms-2482534-supplementary.pdf]

SUPPLEMENTARY INFORMATION

Platelet-Derived Extracellular Vesicles as Lipid Carriers in Severe Allergic Inflammation

Alba Couto-Rodriguez [1], Alma Villaseñor [1,2], Carmela Pablo-Torres [1], David Obeso [1,2], María Fernanda Rey-Stolle [2], Héctor Peinado [3], José Luis Bueno [4], Mar Reaño-Martos [5], Alfredo Iglesias Cadarso [5], Cristina Gomez-Casado [1], Coral Barbas [2], Domingo Barber [1], María M. Escribese [1,†] and Elena Izquierdo [1,*,†]

[1] Departamento de Ciencias Médicas Básicas, Instituto de Medicina Molecular Aplicada (IMMA) Nemesio Díez, Facultad de Medicina, Universidad San Pablo-CEU, CEU Universities, Urbanización Montepríncipe, 28660 Boadilla del Monte, Spain; alba.coutorodriguez@beca.ceu.es (A.C.-R.); alma.villasenor@ceu.es (A.V.); carmela.pablojimenez@ceu.es (C.P.-T.); davidobe12@gmail.com (D.O.); cristina.gomezcasado@med.uni-duesseldorf.de (C.G.-C.); domingo.barberhernandez@ceu.es (D.B.); mariamarta.escribesealonso@ceu.es (M.M.E.)

[2] Centro de Metabolómica y Bioanálisis (CEMBIO), Facultad de Farmacia, Universidad San Pablo-CEU, CEU Universities, Urbanización Montepríncipe, 28660 Boadilla del Monte, Spain; frstolle@ceu.es (M.F.R.-S.); cbarbas@ceu.es (C.B.)

[3] Spanish National Cancer Research Center (CNIO), Molecular Oncology Programme, Microenvironment and Metastasis Laboratory, 28029 Madrid, Spain; hpeinado@cnio.es

[4] Department of Hematology, Hospital Universitario Puerta de Hierro Majadahonda, 28222 Madrid, Spain; jolubuca1898@gmail.com

[5] Department of Allergy and Immunology, Hospital Universitario Puerta de Hierro Majadahonda, 28222 Madrid, Spain; marreano@yahoo.es (M.R.-M.); alfredo.iglesias@salud.madrid.org (A.I.C.)

* Correspondence: elena.izquierdoalvarez@ceu.es; Tel.: +34-91-372-47-00 (ext. 14686)

† These authors contributed equally to this work.

Materials and methods

Detailed description of QCs preparation, instrumental description, data pre-treatment and statistical analysis for metabolomics analysis.

Liquid chromatography coupled to mass spectrometry (LC-MS)

Quality Control Preparation.

A quality control (QC) was set in parallel to the sample preparation. The QC was prepared by aggregating equal volumes (5 µl) of each sample from the study. The QC followed the same procedure applied to the experimental samples. The QC was measured throughout the analysis process to provide a measure of system stability, throughput, and reproducibility of the sample treatment procedure.

LC-MS equipment.

LC−MS analysis was performed on an Agilent HPLC system (1290 Infinity II, Agilent Technologies, Santa Clara, CA, USA), equipped with a degasser, two binary pumps, and a thermostated autosampler coupled to a triple quadrupole-time of flight analyzer mass spectrometer series 6545 (Agilent Technologies, Santa Clara, CA, USA). Ten µl of sample were injected into a Discovery HS C18 column (2.1 mm × 150 mm, 3.0 µm; Supelco, Sigma Aldrich, Germany), with a guard column Discovery® HS C18 (2 cm × 2.1 mm, 3 µm; Supelco, Sigma Aldrich, Germany), both maintained at 40 °C. The flow rate was set at 0.600 ml/min. The elution gradient involved a mobile phase consisting of: (A) MeOH + H2O (1:9) + 0.2 mM NH_4F + 10mM ammonium acetate and (B) ACN:MeOH:Isopropyl Alcohol (IPA) (2:3:5) + 0.2 mM NH_4F + 10mM ammonium acetate. The initial conditions were set at 70% phase B, which increased to 100% phase B in 19 min, followed by re-equilibration for 1 min, and finally it was held for 9 min in initial conditions. The data were acquired using electrospray source ionization (ESI) in both positive and negative ion modes. The capillary voltage was set at 3,500V for ESI+ and 4,000V for ESI−. The drying gas flow rate was 10.5 l/min at 330 °C and the gas nebulizer at 52 psi; fragmentor voltage was 175 V; skimmer and octopole radio frequency voltage (OCT RF Vpp) were set to 65 and 750 V, respectively. Data were collected in the centroid mode at a scan rate of 1.2 Hz. The MS detection window was performed a in full scan from 100 to 1200 *m/z* for both ESI modes. The reference *m/z* ions were purine (*m/z*= 121.0508) and HP-0921 (*m/z*= 922.0097) for ESI+, and TFA NH4 (*m/z*= 119.0363) and HP-0921 (*m/z*= 966.0007) for ESI−. These masses were continuously infused into the system to allow constant mass correction.

Data treatment from LC-MS.

Acquired signals were processed to provide structured raw data in an appropriate format for analysis. Collected data were cleaned together in a single analysis of the background and unrelated ions using Mass Hunter Profinder (B.10.00; SP3, Agilent Technologies) software. The raw data were then filtered by keeping all features that were present after blank subtraction, were detected in >50% of QCs and >75% in the samples and had Relative Standard Deviation (RSD) <30% in the QCs. The rest of the signals were excluded from the analyses. Finally, 242 and 792 chemical entities were obtained that passed LC-MS quality control (QA) in positive and negative ionization, respectively. Missing values were replaced using the k-nearest neighbors (kNN) algorithm using an in-house script developed in Matlab® [1]. The data were normalized using mass spectral total useful signal (MSTUS). Quality of MS analysis and normalization strategy were tested using principal component analysis (PCA) models.

Compound annotation from LC-MS analysis.

Database annotation was performed for statistically significant features (p-value<0.05, p-FDR<0.1) by searching for exact masses in available online databases, such as KEGG, METLIN, LipidMaps, and HMDB, using an advanced online software called CEU Mass Mediator tool [2, 3]. The annotation was performed considering the retention time (RT) gradient, the isotopic pattern and possible adducts. The annotations were confirmed through LC-MS/MS iterative QCs by using a QTOF (model 6545, Agilent). Ions were selected using the narrow *m/z* window (1.3 Da) and 20 eV or 40eV of energy for fragmentation in the quadrupole. Comparison of the structure proposed against the obtained fragments led to the confirmation of the identity.

Gas chromatography coupled to mass spectrometry (GC-MS)

GC-Quadrupole-MS equipment.

GC-MS analysis was performed by a GC system (1890 series, Agilent Technologies, Santa Clara, CA, USA) equipped with auto sampler MultiPurpose Sampler (MPS, Gerstel, Germany) coupled to a mass spectrometer with triple-Axis detector (GC MSD 5977B series, Agilent Technologies, Santa Clara, CA, USA). Two µL of the derivatized sample were injected through a GC-Column DB5-MS (30 m length, 0.250 mm i.d., 0.25 µm film 95 % dimethyl/5 % diphenylpolysiloxane) with an integrated pre-column (10 m J&W integrated with 122-5532G Agilent, Santa Clara, CA, USA) with 1.28 ml/min Helium flow into a Restek 20782 (Bellefonte, PA, USA) glass-wool split liner. Carrier gas (Helium) flow rate was set at

1.28 mL/min and injector temperature at 250 °C. Instrument worked in spitless. The temperature gradient was programmed as follows: the initial oven temperature was set at 60 ºC for 0.5 min, increased to 325 ºC at 10 ºC/min rate (within 37 min), and was held at 325 ºC for 10 min. The total run time was 37.5 min. A 40 ºC period was applied of 5 min before the next injection. Detector transfer line, filament source and the quadrupole temperature were set at 280 ºC, 230 ºC and 150 ºC, respectively. MS detection was performed with electron ionization (EI) mode at -70 eV. The mass spectrometer was operated in scan mode over a mass range of *m/z* 50-600 at a rate of 2.7 scan/s. Several internal standard (IS) injections, a standard mix of alkanes and a mix of fatty acid methyl esters (FAME C8-C30), extraction blanks and 5 QCs samples were injected at the beginning of analysis, following QCs injections every 5 experimental samples and one QC and a blank injection at the end of the worklist.

<u>Data treatment and compound identification from GC-MS data</u>

GC-MS data, peak detection and spectra processing algorithms were applied using Agilent Mass Hunter Software (Agilent, Santa Clara, CA, USA). The overall analytical performance was carefully examined by inspection of total ion chromatograms (TIC) of experimental samples, QC samples, blanks, and internal standard. Automatic mass spectral deconvolution was employed to detect co-eluted compounds using the Unknowns Analysis software version B 10.0 from Agilent. For the identification, the same software allows to perform a library search after the deconvolution process is performed. The assignation is based on spectral comparison and according to their retention indexes (RIs) and retention times (RTs), assigning a match score.

First, a search was conducted in the Fiehn RTL (Retention Time Locked) library, which allowed for direct comparison of retention times. If necessary, through the injection of the FAMES mix, experimental retention time (RT) could be converted to retention index (RI) and compared with the RI values in the library. In the case of missing values, a search was performed in the NIST v.14 library (G1676AA, Agilent, Santa Clara, CA, USA), and, in addition to spectral comparison, the experimental RTs were converted to RIs on the n-alkane scale for comparison with those collected in the library. This conversion was made possible by prior injection of the alkane mixture in the analysis sequence. Integration of the targets and alignment of the drift were performed on MassHunter Quantitative B 10.0 (Agilent, Santa Clara, CA, USA). Raw data filtering was performed by removing those features detected in <50% of all QC samples and with a relative standard deviation (RSD) >30% in QC samples. Finally, 38 metabolites were obtained after QA.

Statistical analysis of metabolomic studies

Multivariate analysis was conducted using SIMCA v.16.0 (Sartorius Stedim Data Analytics). A PCA model was employed to assess data quality and identify patterns in samples, and Partial Least Square Discriminant Analysis (PLS-DA) was performed for each pairwise comparison. The unit variance (UV) and Pareto (Par) scale were used in the negative and positive mode models, respectively. The models were evaluated based on the R2 and Q2 parameters, which represent the classification and prediction capabilities, respectively. Subsequently, univariate analysis was conducted in MATLAB (v.R2018b, MathWorks®, Natick, Massachusetts, USA) to determine the p-value for each compound in the study. After examining the data distribution, pairwise comparisons were performed using Mann-Whitney U test (MWU) with a two-sided p-value < 0.05 and False Discovery Rate (FDR) < 0.1 were considered for the selection of statistically significant differences to avoid missing potential metabolites biomarker candidates as in exploratory studies [4, 5]. The Venny online tool (v. 2.0) was utilized to generate a Venn diagram and the MetaboAnalyst online tool (v. 5.0) [6] was employed to construct heat maps with hierarchical clustering. Pearson distance measures were chosen as the clustering parameters, pathways of the significant identified compounds were determined using IMPaLA (v 13.0) online tool (http://impala.molgen.mpg.de/) and were selected those pathways where 2 or more metabolites were involved with a p-value < 0.05.

References

[1] Armitage, E.G.; Godzien, J.; Alonso-Herranz, V.; López-Gonzálvez, Á; Barbas, C. Missing value imputation strategies for metabolomics data. *Electrophoresis* **2015**, *36*, 3050-3060, 10.1002/elps.201500352.

[2] Gil de la Fuente, Alberto; Godzien, J.; Fernández López, M.; Rupérez, F.J.; Barbas, C.; Otero, A. Knowledge-based metabolite annotation tool: CEU Mass Mediator. *J Pharm Biomed Anal* **2018**, *154*, 138-149, 10.1016/j.jpba.2018.02.046.

[3] Gil-de-la-Fuente, A.; Godzien, J.; Saugar, S.; Garcia-Carmona, R.; Badran, H.; Wishart, D.S.; Barbas, C.; Otero, A. CEU Mass Mediator 3.0: A Metabolite Annotation Tool. *J. Proteome Res.* **2019**, *18*, 797-802, 10.1021/acs.jproteome.8b00720.

[4] Benjamini, Y.; Krieger, A.M.; Yekutieli, D. Adaptive linear step-up procedures that control the false discovery rate. *Biometrika* **2006**, *93*, 491-507, 10.1093/biomet/93.3.491.

[5] Vinaixa, M.; Samino, S.; Saez, I.; Duran, J.; Guinovart, J.J.; Yanes, O. A Guideline to Univariate Statistical Analysis for LC/MS-Based Untargeted Metabolomics-Derived Data. *Metabolites* **2012**, *2*, 775-795, 10.3390/metabo2040775.

[6] Pang, Z.; Chong, J.; Zhou, G.; de Lima Morais, David Anderson; Chang, L.; Barrette, M.; Gauthier, C.; Jacques, P.; Li, S.; Xia, J. MetaboAnalyst 5.0: narrowing the gap between raw spectra and functional insights. *Nucleic Acids Res.* **2021**, *49*, W388-W396, 10.1093/nar/gkab382.

Supplementary figures and tables

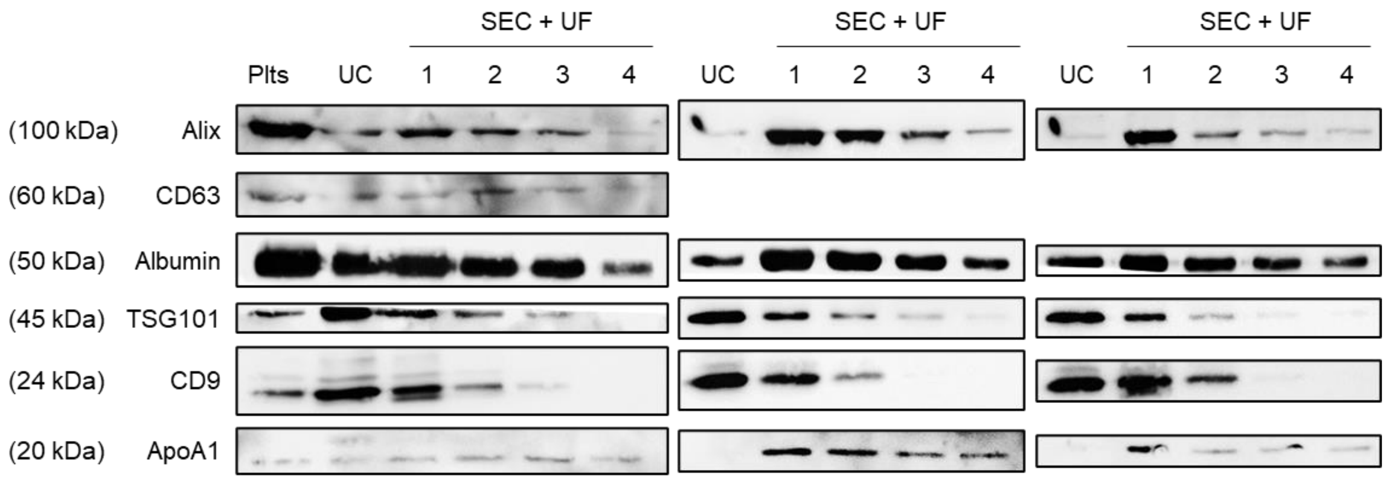

Figure S1. Expression of EV (Alix, CD63, TSG101 and CD9) and non-EV components (albumin and ApoA1) obtained by Western blot in three control subjects. The numbers corresponding to the SEC + UF represent the fractions collected. Platelets (Plts) were used as a control. UC, ultracentrifugation method; SEC + UF, size exclusion chromatography + ultrafiltration method; ApoA1, Apoprotein A1; ALIX, ALG-2 interacting protein X.

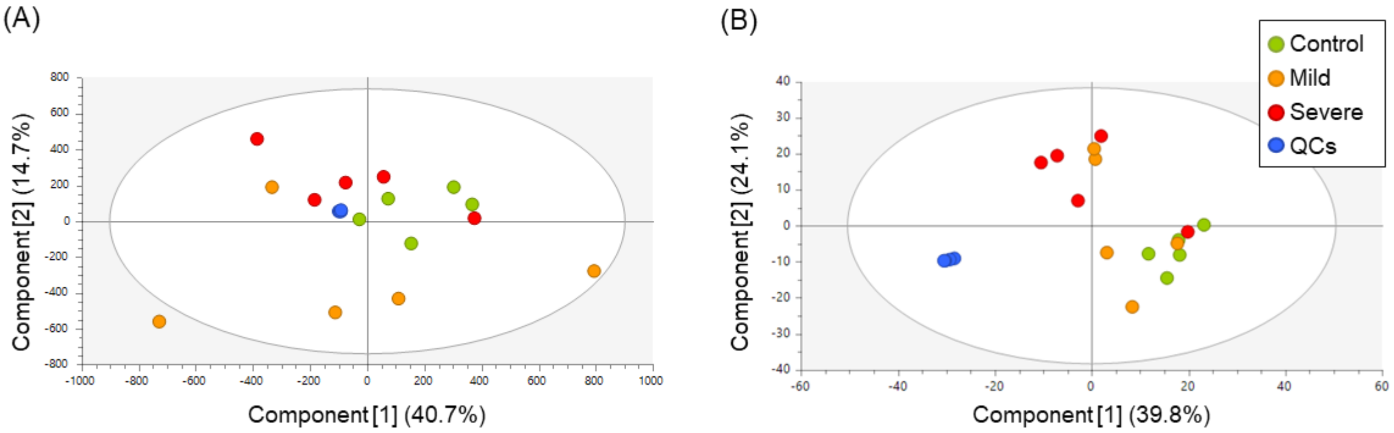

Figure S2. Quality assurance (QA) of the data analysis by principal component analysis (PCA) models of LC-MS from (A) positive and (B) negative mode. Pareto and Univariate scaling were used in positive and negative mode respectively. Samples from control subjects are shown in green, mild allergic patients in orange, severe allergic patients in red, and QCs in blue. QCs=Quality Controls.

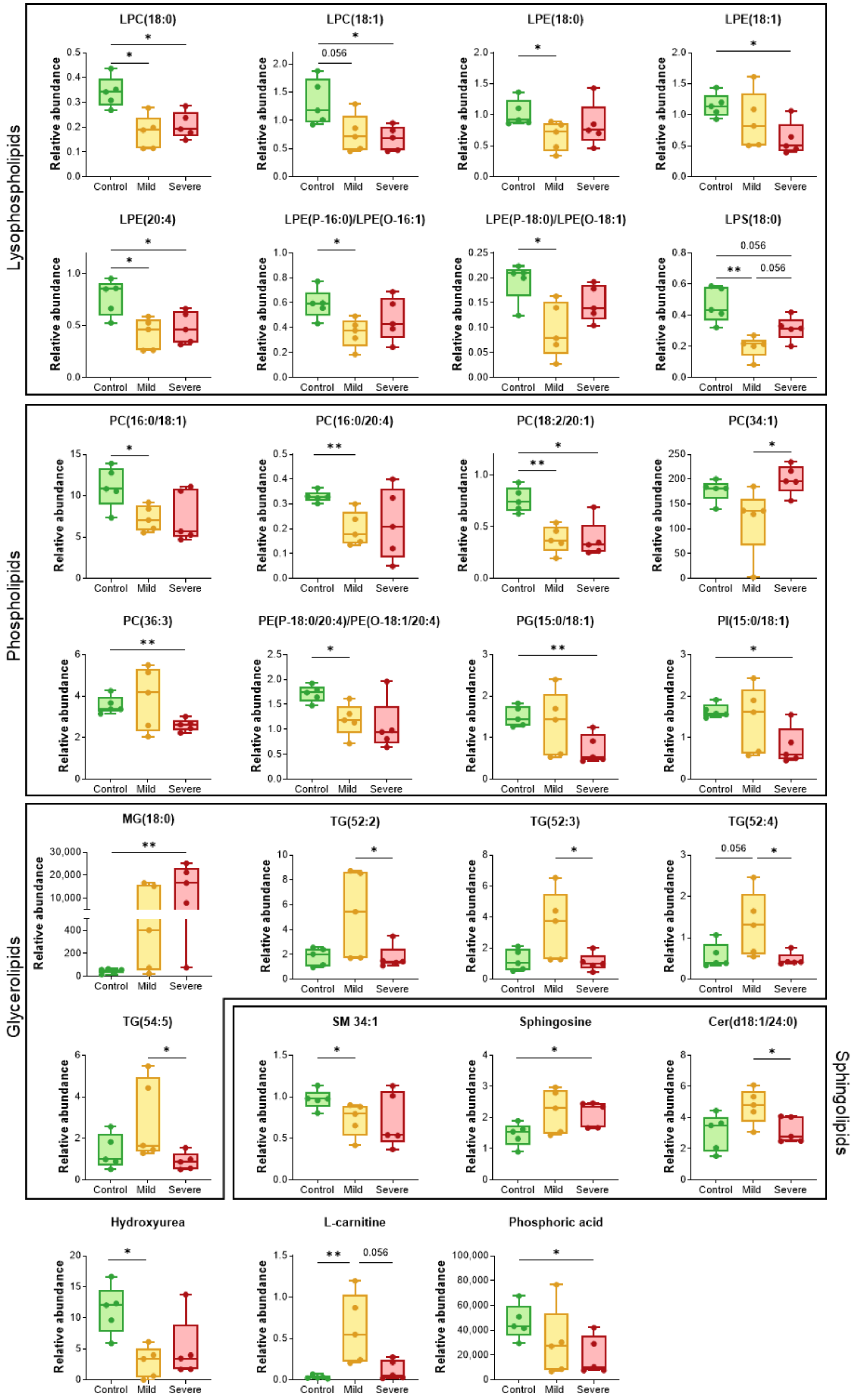

Figure S3. Box and whisker plot representing the relative abundance of the significant identified PL-EVs metabolites between the experimental groups. The central box covers the interquartile range with the median indicated by the line within the box. The whiskers extend to the minimum and maximum values. Mann-Witney U test was used to calculate significant differences. *$p < 0.05$; **$p < 0.01$.

Table S1. Individualized patients' clinical information

								**SPT** (mm)													**Whole blood Hemogram** (x10^9/L)		**PRP hemogram**	
**Patient**	**Sex**	**Age**	**Onset age**	**Smoking**	**Reactions**	**FVC** (%)	**FEV1** (%)	**Ole**	**Phl**	**Cup**	**Pla**	**Cyn**	**Weeds**	**Fra**	**Pro**	**Alt**	**Dpt**	**Dfar**	**Cat**	**Dog**	**PLTs**	**WBC**	**PLTs** (x10^9/L)	**MPV** (fL)
**Control**																								
C-1	F	26		no																	316	7.98	1213	10.00
C-2	M	38		yes																	199	5.85	903	8.40
C-3	F	26		no																	254	5.77	700	8.40
C-4	F	32		no																	188	4.57	361	8.30
C-5	F	30		no																	206	5.14	1164	9.30
C-6	M	24		no																	194	6.85	896	9.00
C-7	F	42		former																	355	7.01	869	8.80
C-8	F	33		no																	258	5.88	887	9.00
**Mild**																								
M-1	M	37	10	no	RC, AS	nv	nv	8x8	13x10	9x6		17x7	4x4	21x20					5x4		235	3.93	983	9.00
M-2	F	36	28	no	RC, AS	na	na	5x5	4x4	5x5	4x4			6x6							240	6.23	1419	10.00
M-3	M	43	36	no	RC, AS	nv	nv	7x7	10x8	8x7	6x6		7x7	10x8							265	7.63	1100	9.60
M-4	F	27	15	no	RC, AS	nv	nv	5x5	5x6	5x5				15x9				7x5	10x10		311	6.54	1310	10.10
M-5	F	22	15	no	RC, AS	nv	nv	9x8	6x6			6x5	6x6	8x7	5x4	6x6			6x7	3x3	263	8.18	670	9.00
M-6	M	50	14	no	RC, AS	na	na	5x5	10x8	12x12	10x8	24x18	3x3		8x8	14x8					334	10.79	1124	8.90
**Severe**																								
S-1	F	53	23	no	RC, AS	nv	nv	10x8	17x12	8x7	10x9	7x7	4x4	8x7			3x3	3x3	6x5	9x9	210	6.35	1085	10.00
S-2	F	28	15	no	RC, AS	62%	60%			5x5							8x5	7x7			313	7.06	1390	9.20
S-3	F	40	27	no	RC, AS	69%	65%		10x11	7x7		4x4									194	6.55	1053	10.00
S-4	F	21	6	no	RC, AS	68%	53%										6x6	11x9	12x8	9x9	326	5.70	1189	8.90
S-5	F	27	8	no	RC, AS	72%	64%			4x4					4x3					7x5	299	7.38	1366	9.30
S-6	F	36	22	no	RC, AS	69%	73%	4x6	5x4	4x3											222	7.63	706	9.00

Note: F: Female. M: Male. RC: Rhinoconjunctivitis. AS: Asthma. FVC: Forced Vital Capacity. FEV1: Forced Expiratory Volume in 1 second. nv: Normal Values (>80%). na: Not Available. SPT: Skin Prick Test. Ole: Olive. Phl: Grass. Cup: Cupressus. Pla: Platanus. Cyn: Cynodon. Fra: Fraxinus. Pro: Profilin. Alt: Alternaria. Dpt: Dermatophagoides pteronyssinus. Dfar: Dermatophagoides farinae. PLTs: Platelets. WBC: White Blood Cells. MPV: Mean Platelet Volume.

Table S2. Statistical analysis of the clinical characteristics of the study subjects.

		Control (n=8)	Mild (n=6)	Severe (n=6)	p-value
		(95% CI) or Freq (%)			
**Demographics (Allergy Service HUPH)**					
**Gender (Female)** F		75%	50%	100%	0.182
**Age (Mean ± SD)** ¶		31.38 ± 6.25	35.33 ± 10.29	34.17 ± 11.44	0.726
**Onset age (Mean ± SD)** ℧			19.67 ± 10.05	16.83 ± 8.57	0.629
**Smoking (Yes)** F		12.5%	0.0%	0.0%	1.000
**Reactions (RC+AS)** F			100.0%	100.0%	1.000
**FVC (%) (abnormal)** F			0.0%	83.3%	0.015
**FEV1 (%) (abnormal)** F			0.0%	83.3%	0.015
**SPT (%) (Positive)** F	**Olive**		100.0%	33.3%	0.061
	**Grass**		100.0%	50.0%	0.182
	**Cupressus arizonica**		83.3%	83.3%	1.000
	**Platanus**		50.0%	16.7%	0.545
	**Cynodon**		50.0%	33.3%	1.000
	**Weeds**		66.7%	16.7%	0.242
	**Fraxinus**		83.3%	16.7%	0.080
	**Profilin**		33.3%	16.7%	1.000
	**Alternaria**		33.3%	0.0%	0.455
	**Dpt**		0.0%	50.0%	0.182
	**Dfar**		16.7%	50.0%	0.545
	**Cat**		50.0%	33.3%	1.000
	**Dog**		16.7%	50.0%	0.545
**PLTs ((Mean ± SD) x 10^9/L)** ¶		246.25 ± 61.95	274.67 ± 39.61	260.67 ± 58.28	0.482
**WBC ((Mean ± SD) x 10^9/L)** ¶		6.13 ± 1.10	7.22 ± 2.28	6.78 ± 0.72	0.370
**PRP hemogram (Transfusion Unit HUPH)**					
**PLTs ((Mean ± SD) x 10^9/L)** ¶		874.13 ± 265.65	1101.00 ± 262.69	1131.50 ± 250.80	0.177
**MPV ((Mean ± SD) fL)** ¶		8.90 ± 0.57	9.43 ± 0.54	9.40 ± 0.49	0.148

Note: ¶: Kruskal-Wallis test. ℧: Mann Whitney test. F: Fisher's exact test. CI: confidence interval. Freq: frequency. F: Female. M: Male. RC: Rhinoconjunctivitis. AS: Asthma. FVC: Forced Vital Capacity. FEV1: Forced Expiratory Volume in 1 second. Abnormal Values for FVC and FEV1 <80%. SPT: Skin Prick Test. Dpt: Dermatophagoides pteronyssinus. Dfar: Dermatophagoides farinae. PLTs: Platelets. WBC: White Blood Cells. MPV: Mean Platelet Volume.

Table S3. Distribution of the patient samples throughout the study.

Subject	Extraction method comparison	PL-EVs Characterization	Metabolomic studies
Control			
C-1		x	x
C-2		x	x
C-3		x	x
C-4	x	x	
C-5	x		x
C-6	x		
C-7		x	
C-8		x	x
Mild			
M-1		x	x
M-2		x	
M-3		x	x
M-4		x	x
M-5		x	x
M-6		x	x
Severe			
S-1		x	x
S-2			x
S-3			x
S-4		x	
S-5		x	x
S-6		x	x
Total Samples	3	16	15

Table S4. Comparison of PL-EVs characteristics obtained by UC and SEC+UF isolation methods.

	UC					SEC+UF					P-value
	N	Min	Mean	SD	Max	N	Min	Mean	SD	Max	
**10^9 particles / mL**	3	6.300	18.900	11.200	27.800	3	22.900	26.500	3.180	29.000	0.750
**µg protein / mL**	3	274.70	411.20	223.80	669.50	3	252.80	497.30	246.90	746.50	0.500
**Mean size** (nm)	3	128.90	172.90	38.74	202.00	3	124.50	128.50	3.49	130.90	0.250
**µg protein / 10^6 particles**	3	10.430	27.890	16.660	43.600	3	9.194	19.590	11.920	32.600	0.750

Note: Paired non-parametric statistical analysis: Wilcoxon matched-pairs signed rank test with Two-tailed P-value. Confidence level: 95%. PL-EVs: Platelet derives Extracellular Vesicles. UC: Ultracentrifugation method. SEC+UF: Size Exclusion Chromatography + Ultrafiltration method. N: Samples number. Min: minimum value. Max: maximum value. SD: Standard Deviation.

Table S5. Comparison of PL-EVs characteristics obtained from control subjects, mild ans severe allergic patients.

	Control					Mild					Severe					p-value
	N	Min	Mean	SD	Max	N	Min	Mean	SD	Max	N	Min	Mean	SD	Max	
**10^9 particles / mL**	6	0.628	0.894	0.377	1.620	6	0.353	0.870	0.518	1.580	4	0.624	1.024	0.311	1.370	0.543
**µg protein / mL**	3	571.68	669.61	160.08	854.34	3	321.21	618.64	329.67	973.11	2	590.98	594.28	4.66	597.57	0.707
**Mean size** (nm)	6	138.20	154.70	15.53	179.60	6	141.70	160.28	12.72	174.40	4	136.60	153.85	16.21	175.60	0.749
**µg protein / 10^6 particles**	3	0.826	0.851	0.023	0.870	3	0.662	0.812	0.132	0.910	2	0.609	0.778	0.239	0.947	0.986

Note: No paired non-parametric statistical analysis: Kruskal-Wallis test. Confidence level: 95%. N: Samples number. Min: minimum value. Max: maximum value. SD: Standard Deviation.

Table S6. Physicochemical properties and analytical parameters of identified significant metabolites from all comparisons and techniques in the study.

Method	Name id	Formula	Adduct	RT (avg)	Mass (avg)	*m/z*	CV% in QCs	HMDB id	CAS#
LC/MS Neg	LPC(18:0)	$C_{25}H_{44}NO_7P$	$M+C_2H_4O_2$	2.26	583.2879	582.2807	13.66	HMDB0011128	4421-58-3
LC/MS Neg	LPC(18:1)	$C_{26}H_{52}NO_7P$	$M+C_2H_4O_2$	2.78	581.3694	580.3622	7.69	HMDB0010385	na
LC/MS Neg	LPE(18:0)	$C_{23}H_{48}NO_7P$	M-H	3.57	481.3221	480.3149	8.24	HMDB0011130	69747-55-3
LC/MS Neg	LPE(18:0)	$C_{23}H_{48}NO_7P$	$M+C_2H_3NaO_2$	3.57	563.3203	562.3131	3.64	HMDB0011130	69747-55-3
LC/MS Neg	LPE(18:1)	$C_{23}H_{46}NO_7P$	M-H	2.89	479.3014	478.2942	10.84	HMDB0011476	na
LC/MS Neg	LPE(18:1)	$C_{23}H_{46}NO_7P$	$M+C_2H_3NaO_2$	2.88	561.3042	560.2970	7.16	HMDB0011476	na
LC/MS Neg	LPE(20:4)	$C_{25}H_{44}NO_7P$	M-H	2.25	501.2859	500.2787	8.85	HMDB0011518	na
LC/MS Neg	LPE(P-16:0)/LPE(O-16:1)	$C_{21}H_{44}NO_6P$	M-H	3.09	437.2911	436.2839	3.16	HMDB0011152	174062-72-7
LC/MS Neg	LPE(P-18:0)/LPE(O-18:1)	$C_{23}H_{46}NO_6P$	2M-H	3.25	926.6144	925.6072	4.69	HMDB0240598	174062-73-8
LC/MS Neg	LPS(18:0)	$C_{24}H_{48}NO_9P$	M-H	2.93	525.3065	524.2993	4.98	HMDB0240606	119786-67-3
LC/MS Neg	PC(16:0/18:1)	$C_{44}H_{80}NO_8P$	M+Cl	9.44	795.5534	794.5462	8.09	HMDB0007971	na
LC/MS Neg	PC(16:0/18:1)	$C_{44}H_{80}NO_8P$	$M+C_2H_4O_2$	9.44	819.5989	818.5917	2.55	HMDB0007971	na
LC/MS Neg	PC(16:0/20:4)	$C_{44}H_{80}NO_8P$	$M+C_2H_4O_2$	7.03	841.5836	840.5764	7.35	HMDB0007983	na
LC/MS Neg	PC(18:2/20:1)	$C_{46}H_{88}NO_8P$	M+C2H4O2-H	11.62	873.6466	872.6394	23.89	HMDB0008144	na
LC/MS Pos	PC(34:1)	$C_{42}H_{82}NO_8P$	M+H	9.48	759.5793	760.5865	0.39	na	na
LC/MS Pos	PC(36:3)	$C_{44}H_{82}NO_8P$	M+H	8.65	765.5607	766.5679	6.99	na	na
LC/MS Neg	PE(P-18:0/20:4)/PE(O-18:1/20:4)	$C_{43}H_{78}NO_7P$	$M+(2^* C_2H_3NaO_2)$	11.40	915.5581	914.5509	3.36	HMDB0005779	144371-69-7
LC/MS Neg	PG(15:0/18:1)	$C_{39}H_{75}O_{10}P$	M-H	6.74	734.5094	733.5022	5.36	na	na
LC/MS Neg	PI(15:0/18:1)	$C_{42}H_{79}O_{13}P$	M-H	6.48	822.5246	821.5174	3.03	na	na
LC/MS Pos	TG(52:2)	$C_{55}H_{102}O_6$	$M+NH_4$	14.69	875.7937	876.8009	5.16	na	na
LC/MS Pos	TG(52:3)	$C_{55}H_{100}O_6$	$2M+NH_4$	14.18	1747.5624	1748.5696	3.58	na	na
LC/MS Pos	TG(52:4)	$C_{55}H_{98}O_6$	M+K	13.76	892.6947	893.7019	5.33	na	na
LC/MS Pos	TG(54:5)	$C_{57}H_{100}O_6$	$M+NH_4$	13.76	897.7792	898.7864	4.14	na	na
LC/MS Neg	SM 34:1	$C_{39}H_{79}N_2O_6P$	M+Cl	7.24	738.5416	737.5344	2.71	na	na
LC/MS Pos	Sphingosine	$C_{18}H_{37}NO_2$	M+H	2.34	299.2824	300.2896	3.07	HMDB0000252	123-78-4
LC/MS Pos	Cer(d18:1/24:0)	$C_{42}H_{83}NO_3$	M+H	12.58	649.6381	650.6453	3.56	HMDB0004956	34435-05-7
LC/MS Pos	L-carnitine	$C_7H_{15}NO_3$	M+H	0.74	161.1056	162.1128	20.99	HMDB0000062	541-15-1
**Method**	**Name**	**Formula**		**RT**	**Exact Mass**	**Product Ion**		**HMDB id**	**CAS#**
GC/MS	MG(18:0)	$C_{21}H_{42}O_4$	nr	24.91	358.5560	401.0000		HMDB0011131	123-94-4
GC/MS	Hydroxyurea	$CH_4N_2O_2$	nr	9.42	76.0550	277.0000		HMDB0015140	127-07-1
GC/MS	Phosphoric acid	H_3O_4P	nr	9.97	97.9952	314.0000		HMDB0001429	7664-38-2

Note: ESI: Electrospray ionisation. POS: positive. NEG Negative. RT: retention time. LPC: Lysophosphatidylcholine. PC: Phosphatidylcholine. LPE: Lysophosphatidylethanolamine. PE: Phosphatidylethanolamine. LPS: Lysophosphatidylserine. PG: Phosphatidylglicerol. PI: Phosphatidylinositol. TG: Triglyceride. MG: Monoglyceride. SM: Sphingomyelin. Cer: Ceramide. nr: not required. na: not available. avg: average. CV: coefficient of variation. QC: quality controls. The confidence level in LC/MS was MS/MS.

Table S7. Changes in the abundance of significant annotated metabolites in all comparisons.

Method	ESI	Name id	Classification	M / C			S / C			M / S		
				p-value	FDR	FC	p-value	FDR	FC	p-value	FDR	FC
LC/MS	Neg	LPC(18:0)	Lysophospholipid	0.016	0.013	0.52	0.016	0.007	0.61	0.548	0.832	1.17
LC/MS	Neg	LPC(18:1)	Lysophospholipid	0.056	0.164	0.58	0.016	0.007	0.52	0.841	0.922	0.89
LC/MS	Neg	LPE(18:0)	Lysophospholipid	0.032	0.013	0.64	0.151	0.216	0.82	0.690	0.882	1.28
LC/MS	Neg	LPE(18:0)	Lysophospholipid	0.016	0.013	0.63	0.095	0.156	0.70	0.841	0.922	1.11
LC/MS	Neg	LPE(18:1)	Lysophospholipid	0.310	0.670	0.79	0.032	0.009	0.53	0.151	0.212	0.67
LC/MS	Neg	LPE(18:1)	Lysophospholipid	0.151	0.545	0.77	0.008	0.007	0.54	0.421	0.823	0.70
LC/MS	Neg	LPE(20:4)	Lysophospholipid	0.032	0.013	0.55	0.032	0.009	0.62	0.548	0.832	1.14
LC/MS	Neg	LPE(P-16:0)/LPE(O-16:1)	Lysophospholipid	0.016	0.013	0.61	0.222	0.296	0.79	0.310	0.673	1.31
LC/MS	Neg	LPE(P-18:0)/LPE(O-18:1)	Lysophospholipid	0.032	0.013	0.49	0.095	0.156	0.77	0.222	0.418	1.56
LC/MS	Neg	LPS(18:0)	Lysophospholipid	0.008	0.013	0.42	0.056	0.116	0.68	0.056	0.112	1.60
LC/MS	Neg	PC(16:0/18:1)	Phospholipid	0.032	0.013	0.64	0.032	0.009	0.62	0.690	0.882	0.96
LC/MS	Neg	PC(16:0/18:1)	Phospholipid	0.032	0.013	0.65	0.151	0.216	0.67	0.841	0.922	1.03
LC/MS	Neg	PC(16:0/20:4)	Phospholipid	0.008	0.013	0.61	0.310	0.389	0.67	1.000	1.000	1.10
LC/MS	Neg	PC(18:2/20:1)	Phospholipid	0.008	0.013	0.50	0.032	0.009	0.50	0.690	0.882	0.99
LC/MS	Pos	PC(34:1)	Phospholipid	0.095	0.216	0.67	0.222	0.749	1.12	0.016	0.021	1.69
LC/MS	Pos	PC(36:3)	Phospholipid	0.841	0.951	1.09	0.008	0.003	0.73	0.421	1.000	0.67
LC/MS	Neg	PE(P-18:0/20:4)/PE(O-18:1/20:4)	Phospholipid	0.016	0.013	0.70	0.151	0.216	0.62	0.421	0.823	0.89
LC/MS	Neg	PG(15:0/18:1)	Phospholipid	0.690	0.839	0.88	0.008	0.007	0.48	0.151	0.218	0.54
LC/MS	Neg	PI(15:0/18:1)	Phospholipid	1.000	1.000	0.88	0.016	0.007	0.49	0.151	0.218	0.55
GC/MS	-	MG(18:0)	Glycerolipid	0.095	0.216	135.88	0.008	0.117	298.44	0.151	0.218	2.20
LC/MS	Pos	TG(52:2)	Glycerolipid	0.222	0.791	2.87	1.000	1.000	0.97	0.032	0.021	0.34
LC/MS	Pos	TG(52:3)	Glycerolipid	0.095	0.216	2.83	0.841	0.965	0.90	0.032	0.021	0.32
LC/MS	Pos	TG(52:4)	Glycerolipid	0.056	0.216	2.34	0.841	0.965	0.84	0.032	0.021	0.36
LC/MS	Pos	TG(54:5)	Glycerolipid	0.222	0.791	2.11	0.310	0.749	0.66	0.032	0.021	0.31
LC/MS	Neg	SM 34:1	Sphingolipid	0.032	0.013	0.75	0.421	0.494	0.74	1.000	1.000	0.98
LC/MS	Pos	Sphingosine	Sphingolipid	0.222	0.791	1.51	0.032	0.005	1.45	1.000	1.000	0.96
LC/MS	Pos	Cer(d18:1/24:0)	Sphingolipid	0.095	0.216	1.56	0.841	0.965	1.05	0.032	0.021	0.67
GC/MS	-	Hydroxyurea	Carboxylic acid derivative	0.016	0.500	0.25	0.071	0.132	0.44	0.635	0.806	1.74
LC/MS	Pos	L-carnitine	Carnitine	0.008	0.100	18.37	0.310	0.749	3.56	0.056	0.112	0.19
GC/MS	-	Phosphoric acid	Non-metal phosphate	0.222	0.875	0.64	0.032	0.117	0.42	0.841	0.946	0.65

Notes: ESI: Electrospray ionisation. Pos: positive. Neg: Negative. C: Control. M: Mild. S: Severe. FDR: False Discovery Rate; FC: Fold Change; LPC: Lysophosphatidylcholine. PC: Phosphatidylcholine. LPE: Lysophosphatidylethanolamine. PE: Phosphatidylethanolamine. LPS: Lysophosphatidylserine. PG: Phosphatidylglicerol. PI: Phosphatidylinositol. TG: Triglyceride. MG: Monoglyceride. SM: Sphingomyelin. Cer: Ceramide. The colors refer to the group in which the significant metabolite is most abundant in each comparison. Blue for a p-value<0.05 and fold change < 1, and red for a p-value<0.05 and fold change > 1. For GC-MS, FDR was < 0.5. The confidence level was MS/MS.

Table S8. Significant biological pathways in which significant metabolites for each comparison are involved.

Pathway	Pathways category	p-value	FDR
**Control vs. Mild**			
**ABC transporters in lipid homeostasis**	**Transport of small molecules**	**4.60E-05**	**9.25E-02**
**ABC-family proteins mediated transport**	**Transport of small molecules**	**1.58E-04**	**1.76E-01**
**Sphingolipid de novo biosynthesis**	**Metabolism**	**4.92E-04**	**2.36E-01**
**Sphingolipid metabolism**	**Metabolism**	**1.70E-03**	**5.05E-01**
Transport of small molecules	Transport of small molecules	1.82E-02	1.00E+00
Signal Transduction	Signal Transduction	3.09E-02	1.00E+00
**Control vs. Severe**			
Golgi-to-ER retrograde transport	Vesicle-mediated transport	9.24E-08	2.15E-04
HDL remodeling	Transport of small molecules	1.60E-07	2.15E-04
Intra-Golgi and retrograde Golgi-to-ER traffic	Vesicle-mediated transport	1.60E-07	2.15E-04
Plasma lipoprotein remodeling	Transport of small molecules	3.13E-07	2.55E-04
G-protein mediated events	Signal Transduction	3.80E-07	2.55E-04
PLC beta mediated events	Signal Transduction	3.80E-07	2.55E-04
Plasma lipoprotein assembly, remodeling, and clearance	Transport of small molecules	7.42E-07	4.27E-04
Opioid Signalling	Signal Transduction	9.88E-07	4.97E-04
Membrane Trafficking	Vesicle-mediated transport	2.50E-06	1.12E-03
**Sphingolipid de novo biosynthesis**	**Metabolism**	**3.63E-06**	**1.38E-03**
PDGFR-beta signaling pathway	Signal Transduction	1.26E-05	2.98E-03
**Phospho-PLA2 pathway**	**Signal Transduction**	**1.76E-05**	**3.93E-03**
**Sphingolipid metabolism**	**Metabolism**	**2.40E-05**	**5.05E-03**
Vesicle-mediated transport	Vesicle-mediated transport	2.51E-05	5.05E-03
COPI-independent Golgi-to-ER retrograde traffic	Vesicle-mediated transport	3.01E-05	5.77E-03
**ABC transporters in lipid homeostasis**	**Transport of small molecules**	**4.60E-05**	**7.40E-03**
Ca-dependent events	Signal Transduction	4.60E-05	7.40E-03
Choline metabolism in cancer	Metabolism	4.60E-05	7.40E-03
Acyl chain remodeling of CL	Metabolism	5.52E-05	8.46E-03
Acyl chain remodelling of PC	Metabolism	6.52E-05	8.46E-03
**Role of phospholipids in phagocytosis**	**Immune System**	**6.52E-05**	**8.46E-03**
Surfactant metabolism	Metabolism of proteins	6.52E-05	8.46E-03
Synthesis of PG	Metabolism	6.52E-05	8.46E-03
Visual signal transduction: Rods	Sensory Perception	6.52E-05	8.46E-03
Glycerophospholipid biosynthesis	Metabolism	7.28E-05	8.74E-03
Signal Transduction	Signal Transduction	7.53E-05	8.74E-03
Visual signal transduction: Cones	Sensory Perception	7.60E-05	8.74E-03
Glutathione redox reactions I	Metabolism	8.76E-05	9.79E-03
Phospholipid metabolism	Metabolism	9.85E-05	1.06E-02
**Fcgamma receptor (FCGR) dependent phagocytosis**	**Immune System**	**1.00E-04**	**1.06E-02**
G alpha (i) signalling events	Signal Transduction	1.04E-04	1.08E-02
**ABC-family proteins mediated transport**	**Transport of small molecules**	**1.58E-04**	**1.59E-02**
Linoleate metabolism	Metabolism	2.29E-04	2.25E-02
Synthesis of PA	Metabolism	2.91E-04	2.73E-02
GPCR downstream signalling	Signal Transduction	3.05E-04	2.79E-02
Synthesis of PC	Metabolism	3.14E-04	2.81E-02
PI Metabolism	Metabolism	3.37E-04	2.95E-02
ESR-mediated signaling	Signal Transduction	3.61E-04	3.09E-02
Signaling by GPCR	Signal Transduction	5.89E-04	4.94E-02
Triacylglycerol biosynthesis	Metabolism	7.44E-04	6.11E-02

Transport of small molecules	Transport of small molecules	8.94E-04	7.19E-02
Fatty acid metabolism	Metabolism	1.43E-03	7.54E-02
Metabolism of lipids	Metabolism	1.50E-03	7.54E-02
Signaling by Nuclear Receptors	Signal Transduction	9.67E-04	7.54E-02
Glycosphingolipid metabolism	Metabolism	1.09E-03	7.54E-02
Signaling by Receptor Tyrosine Kinases	Signal Transduction	1.13E-03	7.54E-02
Glycerophospholipid metabolism	Metabolism	1.22E-03	7.54E-02
**Sphingosine and sphingosine-1-phosphate metabolism**	**Metabolism**	**1.40E-03**	**7.54E-02**
**Arachidonic acid metabolism**	**Metabolism**	**5.14E-03**	**7.97E-02**
**Innate Immune System**	**Immune System**	**5.59E-03**	**7.97E-02**
**Immune System**	**Immune System**	**8.44E-03**	**7.97E-02**
Metabolism	Metabolism	3.06E-02	1.47E-01
Metabolism of proteins	Metabolism of proteins	3.05E-02	1.47E-01
**Mild vs. Severe**			
Insulin resistance	Signal Transduction	3.18E-05	4.27E-02
**Immune System**	**Immune System**	**1.77E-03**	**3.51E-01**
Metabolism of lipids	Metabolism	3.06E-02	1.00E+00

Note: The pathways were ordered from lowest to highest p-value. The pathways mentioned in the manuscript are highlighted in bold. C: Control. M: Mild. S: Severe. FDR: false discovery rate. p-value<0.05
